# Case Report: Bilateral visual loss due to endogenous endophthalmitis following total pancreatectomy with duodenectomy and splenectomy

**DOI:** 10.3389/fsurg.2026.1875699

**Published:** 2026-09-14

**Authors:** Min Gao, Xiaoxi Chen, Hongqiao Gao, Yongsu Ma, Xiaodong Tian, Lanruo Lin, Yinmo Yang

**Affiliations:** 1 Department of Thyroid and Breast Surgery, Peking University First Hospital, Beijing, China; 2 Department of Hepatobiliary and Pancreatic Surgery, Peking University First Hospital, Beijing, China; 3 Department of Ophthalmology, Peking University First Hospital, Beijing, China

**Keywords:** endogenous endophthalmitis, klebsiella pneumoniae, pancreatic diabetes, splenectomy, total pancreatoduodenectomy

## Abstract

This case report describes a patient who developed *Klebsiella pneumoniae* infection and endogenous endophthalmitis following total pancreatectomy with duodenectomy and splenectomy. Despite comprehensive treatment with systemic antibiotics, intravitreal antibiotic injections, and pars plana vitrectomy, the patient's bilateral vision failed to recover. This case highlights the importance of enhancing surgeons' awareness of endogenous endophthalmitis as a rare but severe postoperative complication. Early recognition and timely intervention are crucial for improving patient outcomes.

## 1 Introduction

Endogenous endophthalmitis (EE) is a rare hematogenous infection, accounting for 2%–15% of all endophthalmitis cases. In East Asia, *Klebsiella pneumoniae* accounts for 45.6–60.8% of EE cases (1), which often leads to severe vision loss (66–74.4% end with counting fingers or worse). No clear treatment guidelines exist, but urgent ophthalmic intervention—including vitreous sampling, intravitreal antibiotics, and early pars plana vitrectomy—is required alongside systemic therapy (2).

Unlike previously reported sources of EE (e.g., liver abscess, pneumonia), we describe a patient who developed EE on postoperative day 20 following total pancreatectomy with duodenectomy and splenectomy.

## 2 Case description

A 65-year-old Chinese woman presented with a 5-day history of fatigue and jaundice. On admission, her temperature was 36.4 °C, pulse rate 78 beats/min, blood pressure 135/86 mmHg, and respiratory rate 18 breaths/min. Physical examination revealed icteric skin; the abdomen was soft, without a palpable mass, and no other clinically significant abnormalities were noted. She had no history of diabetes mellitus, cataract, ocular trauma, or other chronic medical conditions. Her past surgical history included laparoscopic appendectomy 2 years earlier. She had no smoking or alcohol use history, and her family history was negative for diabetes mellitus or malignancy. Abdominal computed tomography (CT) revealed a 2.4 × 2.4 cm mass in the pancreatic head, highly suggestive of malignancy, with approximately 180° involvement of the superior mesenteric vein (SMV) and portal vein (PV). Laboratory investigations demonstrated elevated hepatobiliary enzymes (Supplementary Table S1) and a carbohydrate antigen 19-9 (CA 19-9) level of 93.3 U/mL, whereas other tumor markers remained unremarkable.

The patient underwent total pancreatectomy with duodenectomy and splenectomy. Intraoperative exploration confirmed tumor invasion into the SMV and PV, necessitating an artificial vascular bypass. Furthermore, due to severe splenic venous congestion and the anticipated risk of future variceal bleeding, the surgical plan was extended to include a total pancreatectomy and splenectomy. Histopathological examination confirmed a moderately differentiated pancreatic ductal adenocarcinoma (2.3 × 1.7 × 2.4 cm) exhibiting perineural invasion without lymphovascular emboli. The final pathological stage was pT2N0 (AJCC 8th edition). Two abdominal drains were placed at the conclusion of the surgery.

The postoperative course was marked by fluctuating inflammatory markers (Figure 1). Following an initial WBC elevation on postoperative day (POD) 6, the patient developed a spiking fever (39.6 °C) and leukocytosis on POD 15. Blood cultures on POD 18 confirmed *Klebsiella pneumoniae* (sensitive to meropenem and ceftazidime, Supplement Table S2). Despite antibiotic therapy, the patient reported bilateral blurred vision on POD 20. Funduscopic examination revealed bilateral retinal hemorrhages and Roth spots, leading to a diagnosis of localized exudative retinal detachment in the right eye (OD) on the same day. Following recurrent fever and worsening ocular hyperemia, the patient's visual acuity progressively declined in both eyes, prompting consideration of endogenous endophthalmitis on POD 29.

**Figure 1:**
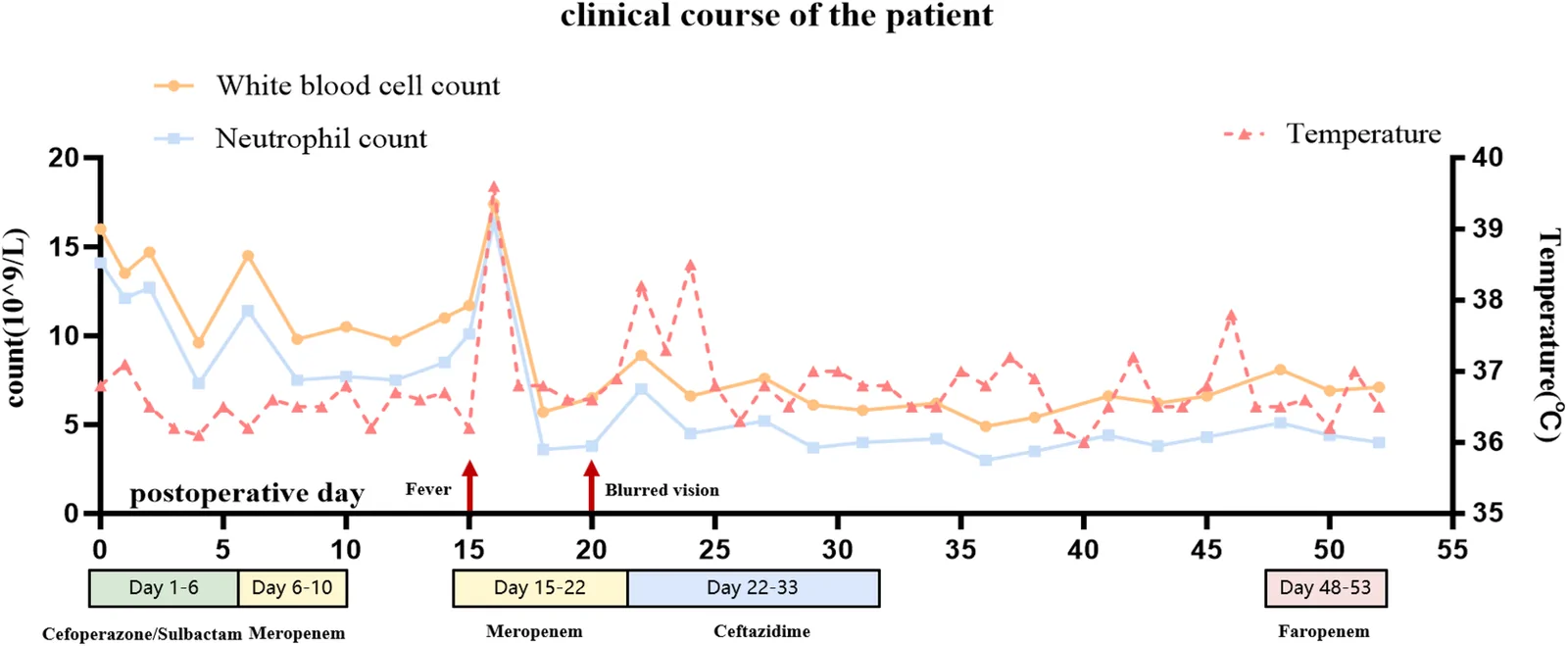
Clinical course of the patient.

Supplementary Table S3 summarizes the chronological clinical course, ocular findings, working diagnoses, treatments, and clinical interpretations. Supplementary Table S4 presents serial visual acuity (VA) and intraocular pressure (IOP) measurements for both eyes. Figure 2 illustrates the chronological progression of fundus and B-scan ultrasonography findings, while Figure 3 presents optical coherence tomography (OCT) images of both eyes at selected time points. Bilateral topical therapy with levofloxacin, prednisolone acetate, and atropine was initiated on POD 27. Intravitreal ceftazidime was administered on POD 29, followed by intravitreal ceftazidime plus amikacin on POD 31; bilateral retrobulbar dexamethasone injections were administered on POD 34, 37, and 38. After initial fluctuations, visual acuity in the left eye (OS) stabilized at approximately 0.4, whereas visual acuity in the right eye (OD) remained severely impaired, with a later recorded value of 0.02. On POD 48, after the development of conjunctival congestion in the left eye, systemic antimicrobial therapy was switched to oral faropenem. The localized exudative retinal detachment remained stable throughout the clinical course, without evidence of progression or spontaneous resolution. After discharge, the patient underwent pars plana vitrectomy (PPV) with silicone oil tamponade in the right eye at another hospital on POD 78, followed by PPV with silicone oil tamponade and intraocular lens implantation in the left eye on POD 92. These vitrectomies were performed primarily to address severe vitreous opacity and associated visual impairment and to reduce the risk of recurrent endophthalmitis. Despite these interventions, no further improvement in visual acuity was observed. During a telephone follow-up conducted 8 months after total pancreatectomy with duodenectomy and splenectomy, the patient reported visual acuity at the counting-fingers level in both eyes; no additional detailed measurements were available.

**Figure 2:**
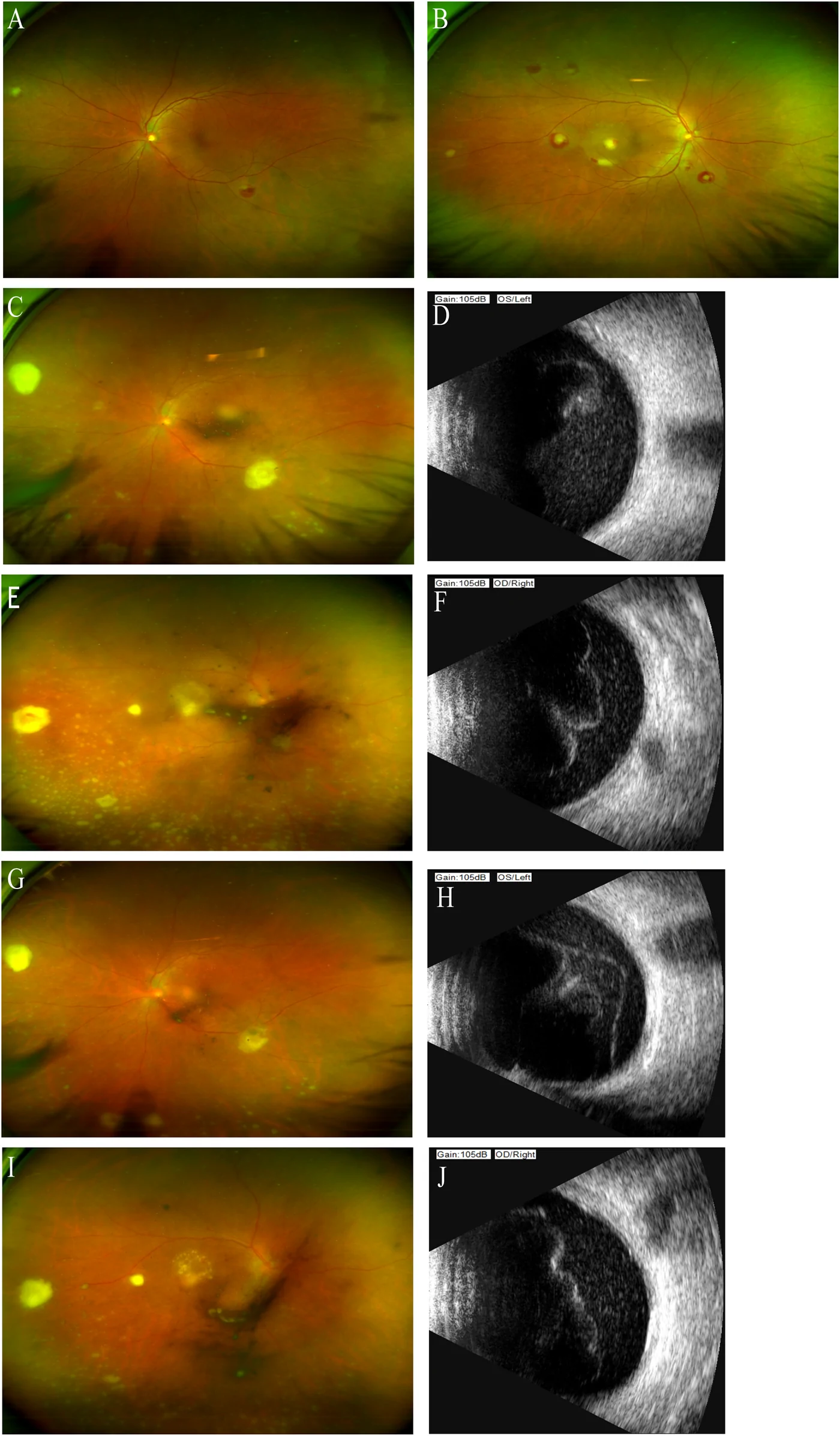
Chronological progression of fundus and B–Scan ultrasonography findings. (A,B) Fundus photographs on postoperative day 20. **(A)** The left eye shows a normal optic disc with distinct margins and normal color, a cup-to-disc (C/D) ratio of 0.3, and an artery-to-vein (A:V) ratio of 2:3. The foveal reflex is absent. A yellowish-white exudative mass is visible in the superonasal retina, accompanied by a Roth spot along the inferotemporal vascular arcade. **(B)** The right eye demonstrates multiple scattered Roth spots across the retina. **(C–F)** Fundus photographs and B-scan ultrasonography on postoperative day 34. **(C,D)** Left eye and **(E,F)** right eye reveal pronounced flocculent vitreous opacities, which are more severe in the right eye, obscuring the view of the fundus. Numerous yellowish-white spherical exudates are present within the preretinal vitreous cavity, along with multiple elevated yellowish-white lesions on the fundus. **(G–J)** Fundus photographs and B-scan ultrasonography on postoperative day 40. **(G,H)** Left eye and **(I,J)** right eye indicate mild improvement in the flocculent vitreous opacities. The preretinal yellowish-white spherical exudates have significantly resolved, and the multiple elevated yellowish-white fundus lesions appear more localized compared to the previous examination. No new infectious lesions are observed.

**Figure 3:**
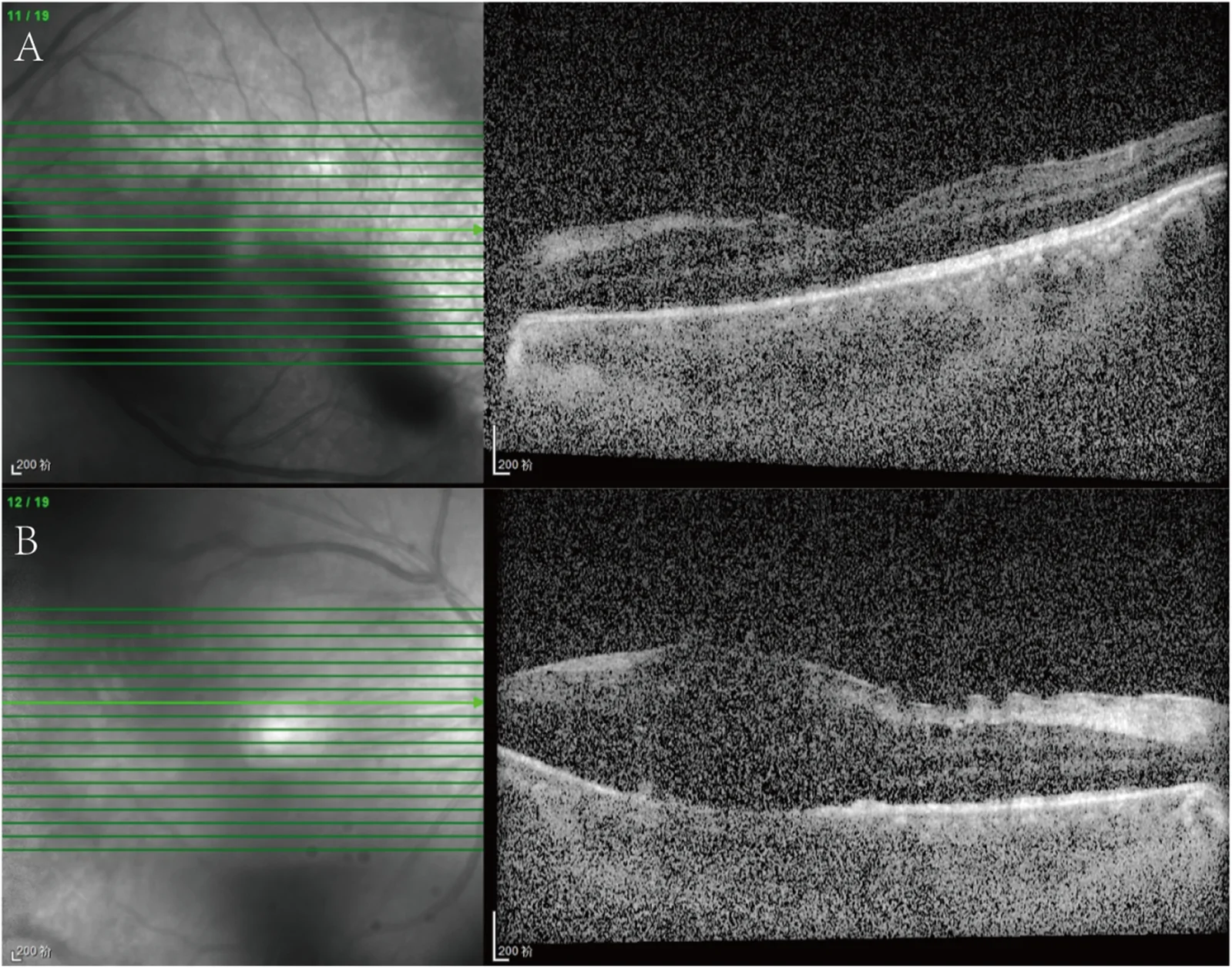
Optical coherence tomography (OCT) images of the patient's eyes at different time points. **(A)** OCT scan of the left eye (OS) on postoperative day (POD) 43, demonstrating a discernable macular architecture accompanied bylocalized intraretinal fluid/edema in the nasal region. **(B)** OCT scan of the right eye (OD) on POD 51, revealing an epiretinal membrane exerting mechanical traction on the macular area, resulting in the complete loss of normal macular foveal anatomy.

## 3 Discussion

This case presents endogenous endophthalmitis (EE) as a systemic manifestation of *Klebsiella pneumoniae* bacteremia following total pancreatectomy with duodenectomy and splenectomy. The patient had no history of diabetes mellitus before surgery, and the preoperative HbA1c level was 5.7% (Supplementary Table S1), supporting the development of new-onset pancreatogenic diabetes after surgery. After oral intake was resumed on POD 11, glycemic management consisted of insulin aspart at 4 IU three times daily before meals and insulin glargine at 8 IU at bedtime. Nevertheless, glycemic control remained suboptimal (Figure 4). Total pancreatectomy causes absolute insulin deficiency and can result in “apancreatic diabetes,” characterized by marked glycemic instability. Surgical stress and parenteral glucose administration may further complicate perioperative glycemic management. Although guidelines recommend relatively permissive glycemic targets of 7.8–13.9 mmol/L (3), persistent hyperglycemia may impair neutrophil chemotaxis and phagocytosis, thereby increasing susceptibility to hematogenous dissemination of *K. pneumoniae*. In this patient, postoperative glycemic dysregulation may have contributed to the development of bacteremia and EE, although a causal relationship cannot be established.

**Figure 4:**
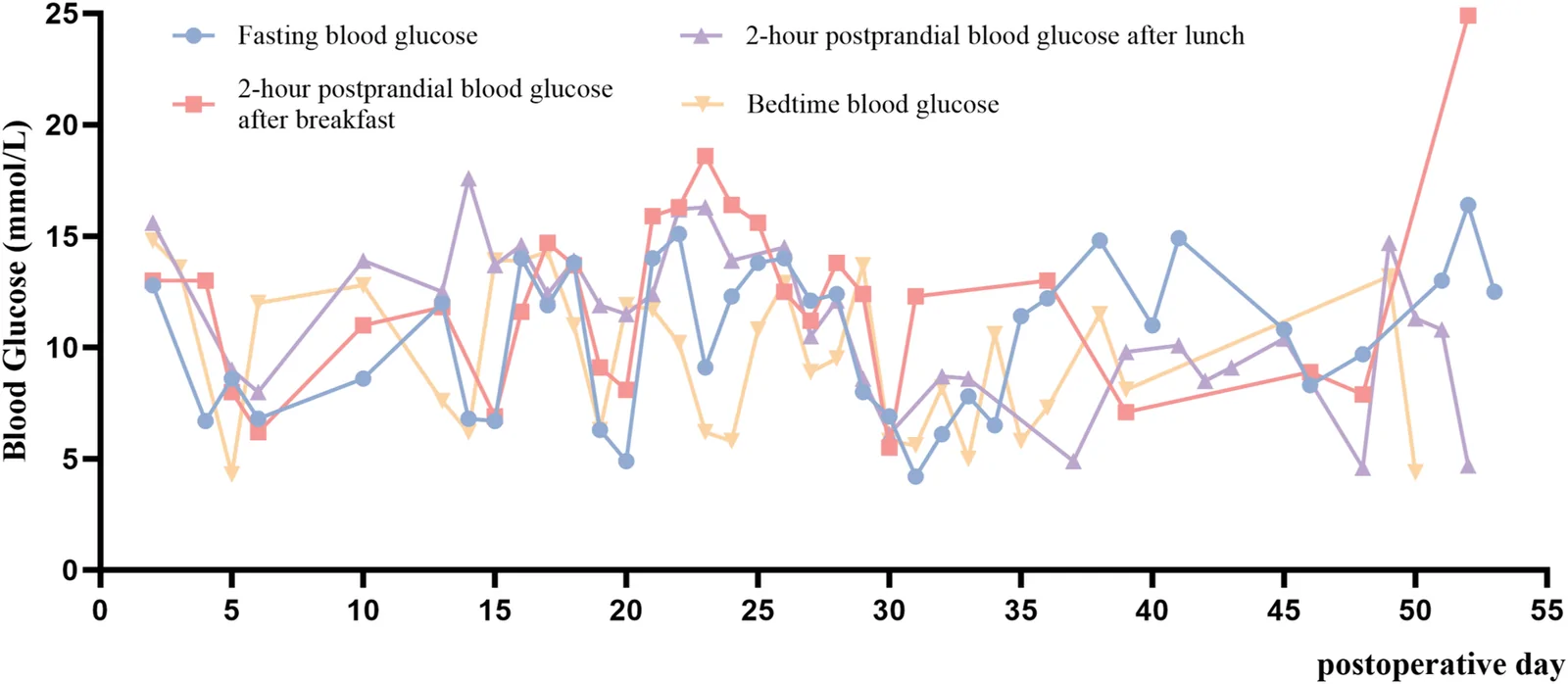
Dynamic changes in blood glucose levels after surgery.

A systematic work-up failed to identify a definite infectious focus. Non-contrast abdominopelvic CT performed on POD15 and POD29 showed no intra-abdominal infection or liver abscess. Blood cultures were obtained twice, on POD15 and POD29; Klebsiella was isolated from the first sample, whereas the repeat culture on POD29 was negative after antimicrobial therapy. Catheter tip and abdominal drainage catheter cultures were negative, likely because antibiotics had already been started. Echocardiography on POD29 excluded infective endocarditis. Serum (1, 3)-β-D-glucan and galactomannan tests were negative, and aqueous humor smear and culture were also negative. Taken together, these findings support a clinical diagnosis of endogenous endophthalmitis in the context of documented Klebsiella bacteremia; however, the ocular pathogen was not microbiologically confirmed, and no genotyping was performed to determine whether the isolate was hypervirulent.

A possible but unproven explanation is postoperative bacterial translocation from the intestinal or biliary tract, facilitated by surgical stress, fasting, prior broad-spectrum antibiotic use, and diabetes-related immune dysfunction. Acute postoperative hyperglycemia may also have transiently impaired blood-ocular barrier integrity and increased susceptibility to hematogenous ocular seeding (4). These mechanisms remain speculative.

Endophthalmitis is defined as a bacterial or fungal infection of the vitreous or aqueous humor. In the absence of preceding ocular disease, surgery, or trauma, an exogenous etiology was effectively excluded in this patient. The diagnosis of EE remains primarily clinical; however, its early manifestations often lack specificity, leading to a high risk of diagnostic delay. A preliminary diagnosis can be established based on ocular symptoms (e.g., hyperemia, ocular pain, blurred vision), specialized ophthalmic examinations (slit-lamp biomicroscopy, funduscopy, and B-scan ultrasonography), and the presence of an extraocular infectious source. Notably, definitive diagnosis should not rely solely on microbial evidence, as intraocular fluid cultures and smears exhibit a relatively low positivity rate, ranging from 35.3% to 54.8% (5). Consequently, clinicians must maintain a high index of suspicion for EE, closely monitor disease progression, and provide comprehensive psychological support and health education. Early diagnosis and prompt intervention are paramount; evidence suggests that early vitrectomy combined with intravitreal antibiotic administration significantly improves visual outcomes (6).

Despite comprehensive pathogenic screening, no intraocular microbiological evidence was obtained to identify the intraocular infection source; thus, the definitive ocular etiology remains strictly presumptive. Although blood cultures isolated *Klebsiella pneumoniae* subsp. *pneumoniae*, the patient exhibited no ocular symptoms at that time; consequently, genotyping was not performed, precluding a definitive microbiological classification. Nonetheless, the clinical constellation of concurrent bacteremia, metastatic endophthalmitis, and poorly controlled pancreatic diabetes raises a high index of suspicion for a putative hypervirulent *K. pneumoniae* (hvKP) strain (7). Typically, hvKP strains harbor specific virulence plasmids encoding a hypermucoviscous phenotype and prominent K1/K2 capsules. These structures mediate resistance to serum killing and phagocytosis, thereby facilitating invasive infections even in mildly immunocompromised hosts. While a definitive confirmation of hvKP would necessitate genomic sequencing (e.g., detecting rmpA or iuc) or evaluation in mouse models (8), the prolonged administration of antibiotics likely prevented any further bacterial recovery from subsequent blood cultures or intraocular fluids after the onset of ocular symptoms, ultimately leaving the microbiological subtyping unfulfilled.

This patient also underwent splenectomy. It is well recognized that patients who have undergone splenectomy have a significantly increased risk of severe complications such as bacteremia and sepsis, and these complications may occur even several years after splenectomy. A review of the literature on endophthalmitis following splenectomy identified only two case reports describing endophthalmitis as a manifestation of postsplenectomy infection (9, 10). Whether the development of endogenous endophthalmitis in this case was related to splenectomy remains unclear.

For patients with EE, early treatment of the primary infection site and screening for systemic metastatic foci are of particular importance. In this patient, systemic infection was successfully controlled with antibiotic therapy, and intraocular inflammation was temporarily managed with intravitreal injections and retrobulbar corticosteroid injections. Unfortunately, the visual impairment resulting from endogenous endophthalmitis did not recover. The patient did not anticipate sudden postoperative vision loss, and this complication caused a severe physical and psychological impact, triggering a marked stress response. This clinical outcome suggests that surgeons should heighten their awareness of rare hematogenous complications.

## Data Availability

The original contributions presented in the study are included in the article/Supplementary Material, further inquiries can be directed to the corresponding author.
