# Supplementary material for: Case Report: Bilateral visual loss due to endogenous endophthalmitis following total pancreatectomy with duodenectomy and splenectomy
Source: Front Surg. 2026 Sep 14;13:1875699. doi: 10.3389/fsurg.2026.1875699 (PMC13617074; doi:10.3389/fsurg.2026.1875699)
Supplement: Supplementary file 1 [file Supplementaryfile1.docx]

**Supplementary**

**Supplementary Table 1. Preoperative laboratory findings**

| **Complete blood count** |  | **Chemistry** |  |  |  |
| --- | --- | --- | --- | --- | --- |
| White blood cells | 4.5 10^9/L | Total protein | 73.6 g/L | GTP | 863 IU/L |
| Red blood cells | 4.05 10^12/L | Albumin | 44.3 g/L | Sodium | 143.62 mmol/L |
| Hemoglobin | 123 g/L | Total bilirubin | 220.0 μmol/L | Potassium | 3.75 mmol/L |
| Platelets | 235 10^9/L | Direct bilirubin | 128.1 μmol/L | Chloride | 105.9 mmol/L |
|  |  | AST | 285 IU/L | Urea | 3.69 mmol/L |
| **Coagulation** |  | ALT | 580 IU/L | Creatinine | 89.9 μmol/L |
| PT-INR | 1.00 | ALP | 462 IU/L | Glucose | 7.03 mmol/L |
| APTT | 33.3 s | LDH | 233 IU/L | HbA1c | 5.7% (4-6) |

*PT-INR* prothrombin time-international normalized ratio, *APTT* activated partial thromboplastin time, *AST* aspartate aminotransferase, *ALT* alanine aminotransferase, *ALP* alkaline phosphatase aminotransferase, *GTP* glutamyl transpeptidase, *LDH* lactate dehydrogenase.

**Supplementary Table 2. Antimicrobial susceptibility profile of Klebsiella pneumoniae isolated from blood culture**

| **Antibiotic** | **MIC (μg/mL)** | **Susceptibility** |
| --- | --- | --- |
| Trimethoprim/sulfamethoxazole | ≥16/304 | Resistant |
| Nitrofurantoin | 64 | Intermediate |
| Levofloxacin | 1 | Intermediate |
| Ciprofloxacin | 1 | Resistant |
| Tobramycin | ≤1 | Susceptible |
| Gentamicin | ≤1 | Susceptible |
| Amikacin | ≤2 | Susceptible |
| Meropenem | ≤0.25 | Susceptible |
| Imipenem | ≤1 | Susceptible |
| Aztreonam | ≤1 | Susceptible |
| Cefepime | ≤1 | Susceptible |
| Clotrimazole | 16 | Intermediate |
| Ceftazidime | ≤1 | Susceptible |
| Cefotetan | ≤4 | Susceptible |
| Cefuroxime axetil | ≥64 | Resistant |
| Cefuroxime (Oral) | ≥64 | Resistant |
| Cefuroxime (Parenteral) | ≥64 | Resistant |
| Cefazolin (Urine) | ≥64 | Resistant |
| Piperacillin/tazobactam | ≤4 | Susceptible |
| Ampicillin/sulbactam | ≥32 | Resistant |

**Supplementary Table 3. Clinical Course and Ophthalmologic Management**

| **POD** | **Key Events** | **Major Ocular Manifestations** | **Working Diagnosis** | **Management** | **Clinical Interpretation** |
| --- | --- | --- | --- | --- | --- |
| 18 | Blood culture positive for *Klebsiella* | No ocular symptoms | Systemic infection | Systemic meropenem |  |
| 20 | First report of blurred vision | Localized exudative retinal detachment in the right eye; bilateral fundus hemorrhages | Bilateral fundus hemorrhages | Systemic anti-infective therapy; close ophthalmic observation | First ophthalmologic consultation. No definite evidence of endophthalmitis was found. |
| 23 | Ophthalmology follow-up | Bilateral fundus hemorrhages; bilateral Roth spots | Infective endocarditis to be excluded | Echocardiography ruled out infective endocarditis | In the setting of *Klebsiella* bacteremia and bilateral fundus hemorrhages with Roth spots, infective endocarditis needed to be excluded; echocardiography showed no supportive evidence. |
| 27 | Marked visual deterioration | Numerous vitreous opacities posterior to the lens | Panuveitis | Bilateral topical treatment: prednisolone acetate eye drops four times daily and atropine ointment once daily | Uveitis with superimposed infection was considered likely; infection focus was sought and systemic anti-infective therapy was continued. |
| 29 | Initial diagnosis of endophthalmitis | Multiple yellow-white elevated lesions in both eyes, enlarged compared with the previous week; abundant yellow-white spherical exudates in the preretinal vitreous; vitreous opacities | Endogenous endophthalmitis | Bilateral intravitreal ceftazidime injection (2 mg) | Given the patient's *Klebsiella* bacteremia, Klebsiella endophthalmitis was considered likely; bilateral intravitreal ceftazidime was administered the same day according to the susceptibility results. |
| 30 |  | No obvious change from the previous day |  | Bilateral topical treatment: levofloxacin eye drops three times daily, prednisolone acetate eye drops three times daily, and ofloxacin ophthalmic ointment at bedtime | The condition was closely monitored, and the patient was re-examined the following day. |
| 31 |  | Fundus examination showed bilateral yellow-white elevated lesions with clearer margins and slightly smaller size than before, but worsening vitreous opacity. |  | Bilateral topical levofloxacin eye drops three times daily and prednisolone acetate eye drops three times daily; bilateral intravitreal injections of ceftazidime (2 mg) and amikacin (400 μg). | The lesions showed partial regression, but the worsening vitreous opacity suggested incomplete control of inflammation. |
| 32 |  | No obvious change compared with the previous day. |  | Bilateral topical levofloxacin eye drops three times daily and prednisolone acetate eye drops three times daily. | The condition was closely monitored. |
| 34 |  | Anterior and posterior segment inflammation, vitreous opacity, and peripheral yellow-white infiltrates. |  | Bilateral retrobulbar dexamethasone injection (5 mg). | Persistent intraocular inflammation. |
| 35 | Aqueous humor was collected for smear microscopy, tNGS*, and bacterial culture. | Same as previous day. |  | Bilateral retrobulbar dexamethasone injection (5 mg). | Subsequent aqueous humor smear microscopy, tNGS, and bacterial culture results were all negative. |
| 37 |  | Yellow-white elevated lesions were better demarcated and more localized, with reduced vitreous opacity. |  | Bilateral retrobulbar dexamethasone injection (5 mg). | The condition had improved compared with previous examinations, and inflammation was under control. |
| 38 |  | No obvious change compared with the previous day. |  | Bilateral retrobulbar dexamethasone injection (5 mg). | The condition remained stable, and treatment was maintained. |
| 50 | Discharged from our hospital | N/A |  | N/A | The patient was discharged in stable condition. |
| 78 | OD PPV |  |  | OD pars plana vitrectomy with silicone oil tamponade. | To address severe vitreous opacity causing visual impairment, and to prevent the recurrence of endophthalmitis. |
| 92 | OS PPV |  |  | OS pars plana vitrectomy with silicone oil tamponade and intraocular lens implantation. |  |

* tNGS, targeted next-generation sequencing.

**Supplementary Table 4. Chronological changes in intraocular pressure and visual acuity during the postoperative period.**

|  | **Intraocular Pressure(mmHg)** | | **Visual Acuity** | | **Notes** |
| --- | --- | --- | --- | --- | --- |
| **POD** | **OD** | **OS** | **OD** | **OS** |  |
| 20 | 17 | 18 | 0.2 | 0.6 |  |
| 23 | 17 | 18 | 0.2 | 0.6 |  |
| 27 | 15 | 18 | CF at 30 cm | 0.25 |  |
| 29 | 15 | 14 | CF at 30 cm | 0.2 |  |
| 30 | 14 | 16 | 0.2 | 0.2 |  |
| 31 | 14 | 13 | CF at 30 cm | 0.15 |  |
| 32 | 15 | 15 | HM | 0.06 |  |
| 33 | 13 | 15 | CF at 30 cm | 0.06 |  |
| 34 | 12 | 13 | CF at 30 cm | 0.12 |  |
| 35 | 11 | 11 | 0.01 | 0.15 |  |
| 36 | 14 | 11 | CF at 30 cm | 0.15 |  |
| 37 | 14 | 16 | 0.01 | 0.3 |  |
| 38 | 14 | 16 | 0.01 | 0.2 |  |
| 39 | 12 | 13 | 0.02 | 0.25 |  |
| 40 | 13 | 14 | 0.02 | 0.4 |  |
| 41 | 13 | 17 | 0.02 | 0.4 |  |
| 43 | 12 | 11 | 0.02 | 0.2 |  |
| 45 | 11 | 13 | 0.02 | 0.25 |  |
| 48 | 14 | 14 | 0.02 | 0.4 |  |
| 50 | N/A | N/A | N/A | N/A | Patient discharged from our hospital. |
| 78 | N/A | N/A | N/A | N/A | After discharge, OD pars plana vitrectomy with silicone oil tamponade was performed at another hospital. |
| 92 | N/A | N/A | N/A | N/A | After discharge, OS pars plana vitrectomy with silicone oil tamponade and intraocular lens implantation was performed at another hospital. |
| 8 months | N/A | N/A | CF (patient-reported)* | CF (patient-reported)* | Follow-up outside our hospital. |

*CF* counting fingers, *HM* hand motions, *OD* oculus dexter (right eye), *OS* oculus sinister (left eye), *POD* postoperative day. *Intraocular pressure measurements were unavailable because the patient was evaluated at an outside hospital. Visual acuity was reported by the patient as counting fingers; the corresponding eye could not be confirmed.
